# Abusive episodes among home-dwelling persons with dementia and their informal caregivers: a cross-sectional Norwegian study

**DOI:** 10.1186/s12877-022-03569-4

**Published:** 2022-11-12

**Authors:** Gunn Steinsheim, Susan Saga, Bonnie Olsen, Helene Kjeka Broen, Wenche Malmedal

**Affiliations:** 1 Department of Public Health and Nursing, Faculty of Medicine and Health Sciences, Norwegian University of Science and Technology (NTNU), Postbox 8905, N-7491 Trondheim, Norway; 2 Department of Family Medicine, Keck School of Medicine, University of Southern California, Los Angeles, USA; 3 Department of Geriatrics, Clinic of Internal Medicine, St Olavs Hospital, Trondheim University Hospital, Trondheim, Norway

**Keywords:** Elder abuse, Dementia, Caregiving, Survey, Norway

## Abstract

**Background:**

Elder abuse is a serious issue with a global prevalence of 15.7% in the community setting. Persons with dementia are at higher risk of elder abuse than the older population in general. With a high and increasing prevalence of dementia this issue cannot be neglected. Hence, the aims of this study were 1) to describe the proportion of abusive episodes among home-dwelling persons with dementia and their informal caregivers, and 2) to explore differences between informal caregivers who have reported committing and not committing abusive acts.

**Methods:**

A cross-sectional survey was conducted among informal caregivers of home-dwelling persons with dementia in Norway from May to December 2021 with a total of 549 participants.

**Results:**

Two-thirds of informal caregivers had committed at least one abusive episode toward the person with dementia in the past year (63.5% psychological abuse, 9.4% physical abuse, 3.9% financial abuse, 2.4% sexual abuse, 6.5% neglect). One-third of informal caregivers had experienced aggression from the person with dementia (33.9% psychological abuse, 7.8% physical abuse, 1.1% financial abuse, 1.4% sexual abuse). Tests for independence showed that the risk of abusive episodes from informal caregivers toward persons with dementia was higher when the informal caregiver was a spouse/partner of the person with dementia and if they experienced aggression from the person with dementia.

**Conclusions:**

The results demonstrate that a majority of informal caregivers commit some form of abusive episodes, and episodes that fall within the scope of psychological abuse are most frequent. This study expands knowledge about elder abuse among home-dwelling persons with dementia. Increased understanding of the dynamics of abuse is essential to be able to reduce risk and prevent abuse.

## Introduction

The World Health Organization (WHO) classifies elder abuse as an important public health issue, defining it as “a single or repeated act, or lack of appropriate action, occurring within any relationship where there is an expectation of trust which causes harm or distress to an older person” [1]. Elder abuse can be divided into five subtypes: physical, psychological/emotional, sexual, financial, and neglect. In a review from 2017, the global prevalence rate of elder abuse in the community setting was estimated to be 15.7% [2], and it is expected that the rates are higher among persons with dementia. In the same review, the pooled prevalence estimates for the subtypes were 11.6% for psychological abuse, 2.6% for physical abuse, 6.8% for financial abuse, 0.9% for sexual abuse, and 4.2% for neglect.

In Norway, the only prevalence study of elder abuse among home-dwelling older persons found a prevalence of elder abuse between 5.2 and 7.2% in the past 12 months [3], but this study excluded persons with severe cognitive impairment and dementia. There is strong evidence for cognitive impairment being a risk factor for elder abuse [4, 5] with odds ratios ranging from 1.2 to 6.24 in the UK and US compared to older persons without cognitive impairment [6]. Therefore, it is expected that the prevalence of elder abuse among persons with dementia will be higher than that found by Sandmoe et al. [3]. Other risk factors for elder abuse related to the older person include functional dependency and poor physical or mental health [4, 5]. Related to the informal caregiver (ICG), burden/stress, mental illness and psychological problems [5] such as being more anxious or depressed [7, 8] are known risk factors for committing elder abuse. Behavioral and psychological symptoms of dementia (BPSD) are both associated with caregiver burden/stress [9] and elder abuse [10–12]. Several studies have also found an increased risk of abuse if the ICGs experience aggression or violent behavior from the person with dementia [7, 8, 13, 14].

In 2015, Alzheimer’s Disease International estimated a prevalence of 54.27 million persons living with dementia in 2020 [15]. Gjøra et al. [16] estimated that of a total population of 5.37 million, there were 101,118 persons with dementia in Norway in 2020 (1.88%) and that the prevalence will be more than doubled by 2050. A majority of persons with dementia in Norway live at home [16]. Health and care services available for home-dwelling persons with dementia in Norway include home nursing services and homecare services such as practical help, along with other services including daycare activities, and dementia education for both themselves and their ICG [17]. Home-dwelling persons with dementia are mainly supported by the ICG, particularly family caregivers who co-reside with the person with dementia [18]. When a dementia diagnosis is given, an estimated 60 to 80 h of informal help are provided per month and up to 160 h per month before the person with dementia arrives at a nursing home, which corresponds to working full time [19].

The COVID-19 pandemic resulted in a lockdown in Norway in March 2020, with social distancing as one of the main strategies. Because of the lockdown, many services for persons with dementia were reduced or canceled. A study from the first two months of lockdown in Norway found that formal care provided for persons with dementia on average decreased by 20.5 h per month, while informal care almost doubled [20]. Both the decrease in formal care and the increase in informal care were more pronounced when the ICG co-resided with the person with dementia. A recent Norwegian study showed that BPSD increased at the beginning of the COVID-19 pandemic, especially for depression and psychotic symptoms [21]. Worse BPSD is associated with an increased risk of abusive episodes [10–12]. Also, a recent study found that elder abuse among home-dwelling older persons increased significantly in the US during the COVID-19 pandemic [22].

Relatives and other ICGs provide most of the care and are an important support for persons with dementia. Although there are several positive aspects of caring for someone who has dementia [23], it may also be challenging. Caregiver distress or a high burden is associated with negative health outcomes for the person with dementia, including worsening of BPDS [9], which can result in an even higher caregiver burden and an increased risk of abusive episodes. Although several previous studies have researched elder abuse among home-dwelling persons with dementia [6, 24], this is the first study on this topic conducted in a Norwegian context. Because cultural differences affect elder abuse [2], it is important to have data from all around the world, including Norway. Also, to our knowledge, this is the first study concerning home-dwelling persons with dementia and their ICGs which includes all subtypes of abuse. To measure abuse in this setting is challenging. In this study, we have measured events and actions that we have defined as “abusive episodes” which fall under the different subtypes of elder abuse. We also use the term “abuse” when referring to abusive episodes within subcategories of elder abuse. It is difficult to determine the severity of the episodes and see the context of their circumstances based on the answers from an anonymous questionnaire. Our intention is to raise awareness among healthcare professionals and policymakers that abusive episodes are happening so that both persons with dementia and, especially, ICGs can get the support they need to prevent elder abuse. The primary objectives of the present study were (1) to describe the proportion of abusive episodes among home-dwelling persons with dementia and their ICGs and (2) to explore differences between ICGs who report or do not report abusive episodes, focusing on ICG characteristics, their relationship with the person with dementia, and whether they have experienced aggression from the person with dementia.

## Methods

This is a cross-sectional study where data were collected through anonymous self-reported pen-and-paper questionnaires. The present study is part of a larger study investigating the role of caregiver burden in the extent of elder abuse among home-dwelling persons with dementia [25]. This study was conducted in accordance with STROBE guidelines for cross-sectional studies [26].

### Setting and participants

The study was conducted among ICGs of home-dwelling persons with dementia in Norway with a recruitment period from May to November 2021. Inclusion criteria required participants to be an adult spouse, cohabitant, partner, relative, or friend caring for someone who has dementia currently living at home and who had personal contact with the person with dementia at least once a week. If there were several ICGs, it was preferred—but not required—that the ICG who provided the most care for the persons with dementia participated.

### Data collection

Recruitment was carried out using three different strategies. Firstly, questionnaires were mailed directly to ICGs registered in the Norwegian Registry of Persons Assessed for Cognitive Symptoms (NorCog) who had consented to be contacted about relevant research projects. Secondly, ICGs were recruited in collaboration with homecare services and/or dementia/memory teams in municipalities and local dementia associations (organized by the Norwegian Health Association) across Norway. Thirdly, ICGs directly contacted the researchers to participate in the study after they had read information about the study through various media or social media channels or partner organizations. The collaborating municipalities and local dementia associations were a convenience sample that effectively represented all regions of Norway, ensuring the geographical diversity of study participants.

All participants received an envelope containing the questionnaire, a combined information letter and consent form describing the project to the ICG, an information letter adapted for the person with dementia, and a stamped return envelope. A total of 2,000 questionnaires were printed and distributed through the three strategies detailed above.

### Measures

#### Demographics variables

The questionnaire contained questions about the ICG’s age, gender, educational level, region of residence, employment status, and relationship with the person with dementia. In addition, questions about the person with dementia’s age, gender, and dementia diagnosis were included.

#### Abusive episodes

To measure abusive episodes within all five subtypes of abuse, we primarily drew items in the questionnaire from two Norwegian cross-sectional studies of elder abuse in nursing homes and among home-dwelling, cognitively healthy, older persons, respectively [3, 27]. These questionnaires cover all five subtypes of abuse and are tested on Norwegian samples, making them uniquely appropriate for this study. We adapted the wording of the questions slightly to suit the study setting. The resulting questionnaire in the present study contained 27 items measuring abusive episodes.

All items are presented in Table 2. Psychological abuse consisted of five items, three from Botngård et al. [27] and two (“Repeatedly ignored the person with dementia …” and “Prevented the person with dementia from meeting …”) from Sandmoe et al. [3]. Physical abuse consisted of nine items, eight from Botngård et al. [27] and one (“Attacked/injured the person with dementia physically in another way”) from Sandmoe et al. [3]. All three items within the financial abuse category were derived from Sandmoe et al. [3]. The subcategory neglect consisted of seven items, four from Botngård et al. [27], two (“Abandoned the person with dementia …” and “Not given the person with dementia enough help …”) from Sandmoe et al. [3], and one item regarding depriving the person with dementia of assistive devices derived from Conrad et al. [28]. Sexual abuse consisted of three items. We could not find any surveys where ICGs were asked about sexual abuse against someone who has dementia, and none of the questions from the studies mentioned above were considered to fit the context of the present study. Therefore, the three items measuring sexual abuse were created for this study. The items were created based on qualitative studies of ICGs of persons with dementia and sexuality [29–31] and through consulting experienced employees at a regional resource center for dementia who had experience in guiding ICGs.

In addition to questions about perpetrating abusive episodes, the ICGs were asked if the person with dementia had performed similar acts toward them within each subtype of abuse. These acts from the person with dementia toward the ICG are referred to as aggression in this article.

### Questionnaire development

The questionnaire was developed in collaboration with the Norwegian Health Association, the Norwegian Pensioners’ Association, the Norwegian Alliance for Informal Carers, and St. Olavs Hospital in three steps. Firstly, the questionnaire was adjusted according to feedback from representatives from the partner organizations. Secondly, individual cognitive interviews [32] about the questionnaire were conducted with ten persons with experience as ICGs of persons with dementia to ensure the quality of the questionnaire. Their feedback only recommended minor adjustments and the questionnaire was adapted accordingly. Thirdly, a small pilot test was carried out with ICGs who met the study inclusion criteria. The intention was to estimate the time to complete the questionnaire and to correct errors, miswordings, ambiguities, and misunderstandings related to questions, answers, and instructions. The participants received instructions, the questionnaire, and a form where they could write feedback related to the questionnaire. Of the 15 pilot questionnaires distributed, seven were returned. The results of the pilot informed minor changes to the questionnaire, including rewording two questions, adding “not applicable” as a response alternative to some questions, and highlighting some of the text in the instructions.

### Ethical considerations

The study focuses on negative aspects of the role of the ICG, which could cause discomfort to the participants. Therefore, written information clearly stated that participation in the study was voluntary and that answers would be anonymized. In addition, the information letter contained information about two helplines, the Dementia Line and Protective Services for the Elderly, in case the ICG needed someone to talk to after participation. The questionnaire and study information were designed in collaboration with user organizations and ICGs caring for someone who has dementia intentionally to minimize discomfort.

In the survey, the ICGs were asked to provide information related to the person with dementia and his/her health. Depending on the degree of cognitive impairment, some persons with dementia are able to give informed consent while others are not. The ICGs were encouraged to inform the person with dementia about the study, and a letter describing the project adapted for the person with dementia was distributed along with the questionnaire. In the event of known inability to consent, the ICGs were encouraged to assess whether the person with dementia would have consented to participate before dementia occurred.

The study was approved by the Regional Ethics Committee for Medical Research (REC) in Mid-Norway (#153444).

### Statistical analysis

Statistical analysis was conducted using Stata Statistical Software: Release 17. No replacements were made for missing data. Ages of ICGs and persons with dementia are presented as mean values and standard deviations (SD). Categorical variables of characteristics of ICGs and persons with dementia and aggression from the person with dementia are described using frequencies and proportions. The occurrence of abusive episodes committed by ICGs are described using proportions in percentages. We created bivariate variables for all subtypes and overall abusive episodes with the categories “abuse” (one or more episodes in the past 12 months) and “no abuse” (never in the past 12 months or not applicable) to calculate proportions of abusive episodes. This way of dichotomizing is consistent with methods in previous studies [3, 27]. For sexual abuse and neglect, ICGs answering “not applicable” were excluded before the proportion of abuse was calculated. Because of the difficulties in determining the severity, intention behind and outcome of the different abusive episodes, we have treated all abusive episodes equally in the analyzes. We present frequencies and proportions for all items included under abusive episodes in the questionnaire to increase transparency for readers. Further, we have chosen to set the criterion for “abuse” as at least one abusive episode in the last year in the association analyzes. Using Pearson’s chi-squared test, the bivariate variables of psychological, physical, and overall abusive episodes were tested for associations against ICGs’ gender, level of education, their relationship with the person with dementia, and psychological, physical, and overall aggression from the person with dementia toward the ICG. If the expected frequencies were below five in any of the tests, Fisher’s exact test was used. The significance level was set to 0.05.

## Results

Of the 2,000 questionnaires, 1,516 were distributed to ICGs. A total of 549 participants responded from May to December 2021, giving a response rate of 36.2%. Of those, nine were excluded before analysis because the ICG reported that the person with dementia was admitted to a nursing home or deceased. ICGs from all regions of Norway were represented in the sample. A detailed description of ICGs’ and persons with dementia’s demographic characteristics is shown in Table 1.

**Table 1:** Demographic characteristics of ICGs and persons with dementia

Variable		n (%)	Mean (SD)	N^a^
ICG				
Age (21–93)			67.4 (11.77)	532
Gender	Male	169 (31.8)		532
	Female	363 (68.2)		
Relationship with PWD	Spouse/cohabitant/partner	338 (63.1)		536
	Child	173 (32.3)		
	Child-in-law	2 (0.4)		
	Sibling	6 (1.1)		
	Other	17 (3.2)		
Co-resides with PWD	Yes	348 (65.0)		535
Highest level of education	Primary school	52 (9.8)		533
	High school	127 (23.8)		
	Vocational school	128 (24.0)		
	University/college ≤4y	116 (21.8)		
	University/college >4y	110 (20.6)		
Region of residency	Oslo and Viken	167 (31.4)		532
	Innlandet	46 (8.7)		
	Agder and South Eastern Norway	85 (16.0)		
	Western Norway	65 (12.2)		
	Trøndelag	102 (19.2)		
	Northern Norway	67 (12.6)		
Employment status	Full-time employment (incl. full-time studies)	145 (27.4)		529
	Working part-time (incl. part-time studies)	50 (9.5)		
	Not working (unemployed, retired, disabled, etc.)	334 (63.1)		
PWD				
Age (53–99)			78.9 (7.77)	536
Gender	Male	279 (52.1)		536
	Female	257 (47.9)		
Residency area	Big city	52 (9.7)		537
	Suburbs or outskirts of a big city	82 (15.3)		
	Town or small city	174 (32.4)		
	Country village	125 (23.3)		
	Farm or home in the countryside	104 (19.4)		
Dementia diagnosis	Alzheimer’s	237 (44.4)		534
	Unspecified dementia/other dementia	28 (5.2)		
	Mixed dementia diagnosis	23 (4.3)		
	Lewy body dementia	29 (5.4)		
	Vascular dementia	49 (9.2)		
	Frontotemporal dementia	54 (10.1)		
	Diagnosis is ongoing	25 (4.7)		
	Don’t know	89 (16.7)		
Duration of dementia symptoms	≤2 years	115 (21.4)		538
	>2–4 years	200 (37.2)		
	>4 years	223 (41.4)		

*PWD* Person with dementia

^a^N varies due to missing data

Frequencies and proportions of each item related to abusive episodes are presented in Table 2. Overall, 66.3% of ICGs reported that they had committed at least one abusive episode toward the person with dementia in the past year. When divided into subtypes of abuse, 63.5% of ICGs reported at least one episode within the scope of psychological abuse, 9.4% within physical abuse, 3.9% within financial abuse, 2.4% within sexual abuse, and 6.5% within neglect.

**Table 2:** Proportions of abusive episodes in the past year, as reported by ICGs

Type of abuse		ICG self-reported incidence rate in the past 12 months (%)						
		N^a^	NA^b^	Never	Once	2–5 times	6–10 times	>10 times
Psychological abuse	Shouted or yelled at PWD	534		45.1	7.9	26.2	9.6	11.2
	Made humiliating and critical remarks to PWD	534		66.5	7.5	17.8	5.4	2.8
	Threatened PWD	536		87.7	4.9	5.8	1.3	0.4
	Repeatedly ignored PWD in such a way that he/she may feel inferior	535		85.0	5.6	7.3	1.5	0.6
	Prevented PWD from meeting others/restricted freedom of movement	536		98.3	0.6	0.6	0.6	0.0
Physical abuse	Pushed, grabbed, or pinched PWD	537		93.7	1.9	3.5	0.6	0.4
	Pulled his/her hair or kicked PWD	537		99.4	0.4	0.2	0.0	0.0
	Threw objects at PWD	538		99.6	0.4	0.0	0.0	0.0
	Beat PWD	538		98.7	0.9	0.4	0.0	0.0
	Bullied/harassed PWD	533		98.5	0.0	1.5	0.0	0.0
	Attacked/injured PWD physically in another way	532		99.1	0.4	0.6	0.0	0.0
	Omitted giving PWD necessary medication	530	39.8	57.7	0.8	1.3	0.0	0.4
	Gave the PWD more medicine than necessary	530	39.1	60.8	0.2	0.0	0.0	0.0
	Postponed giving the PWD his/her medication without this being necessary	530	40.0	60.0	0.0	0.0	0.0	0.0
Financial abuse	Persuaded/pressured PWD to give up/transfer money, valuables, or property to you/others	533		99.4	0.4	0.2	0.0	0.0
	Spent PWD’s money beyond what you had agreed on	536		99.3	0.0	0.4	0.4	0.0
	Prevented PWD from having access to their money, valuables, or property in the way they wanted	534		96.4	0.7	2.1	0.0	0.7
Sexual abuse	In connection with intercourse or other sexual acts, been in doubt whether PWD wanted this contact	533	72.8	25.7	0.6	0.9	0.0	0.0
	Carried out intercourse or other sexual acts even if PWD expressed that they did not want it	532	70.3	29.3	0.2	0.2	0.0	0.0
	Responded to sexual approaches from PWD even if the relationship indicates that this was inappropriate	533	72.8	26.6	0.2	0.4	0.0	0.0
Neglect	Let the PWD wait for help longer than necessary	530	34.0	65.3	0.2	0.6	0.0	0.0
	Omitted to help the PWD with oral care/dental care	530	61.1	37.9	0.2	0.2	0.2	0.4
	Omitted to give the PWD enough food and/or liquids	530	37.9	61.9	0.2	0.0	0.0	0.0
	Did not change the incontinence pad of the PWD	528	74.6	24.8	0.4	0.0	0.0	0.2
	Abandoned the PWD without giving him/her the necessary help	530	35.3	64.0	0.2	0.6	0.0	0.0
	Did not give the PWD enough help with personal hygiene and appropriate attire	530	43.0	55.7	0.2	0.6	0.4	0.2
	Prevented the PWD from wearing glasses, hearing aids, dentures, walkers, wheelchairs, or other aids he/she needs	530	41.7	58.3	0.0	0.0	0.0	0.0

*PWD* Person with dementia

^a^N varies due to missing data. ^b^*NA* not applicable

ICGs were asked to report aggression from the PWDperson with dementia toward themselves within each subtype of abuse. Although the majority of ICGs reported no PWD aggression, all subtypes except neglect were reported. Aggression within the scope of psychological abuse (33.9%) was most common. Table 3 gives a complete overview of the frequencies and proportions within the different subtypes. Overall, 35.3% of the ICGs reported PWD aggression from the person with dementia within at least one subtype of abuse.

**Table 3:** Frequency and proportions of aggression from the person with dementia

Type of abuse	N^a^	No aggression n (%)	Aggression n (%)
Psychological	528	349 (66.1)	179 (33.9)
Physical	524	483 (92.1)	41 (7.8)
Financial	529	523 (98.9)	6 (1.1)
Sexual	508	501 (98.6)	7 (1.4)
Neglect	515	515 (100.0)	0 (0.0)

^a^N varies due to missing data

As shown in Tables 2 and 3, financial abuse, sexual abuse, and neglect had the lowest frequencies. These subtypes were therefore excluded from the comparative analysis. Thus, only psychological abuse, physical abuse, and overall abuse were included. The results are found in Tables 4 and 5. There were no significant differences in the proportions of abusive episodes of any type related to ICGs’ gender. There was a significant association between physical abuse and ICGs’ educational level such that those with higher levels of education were more likely to commit an act of physical abuse. There was also an increasing proportion of psychological and overall abusive episodes among ICGs with a higher level of education, but the associations were not significant (*p* = 0.164–0.233). Spouses and partners reported significantly higher proportions of abusive episodes compared to other ICGs. There were significant positive associations between aggression from the person with dementia and abusive episodes committed by ICGs toward the person with dementia, with two exceptions; overall and psychological aggression were not significantly associated with ICGs committing physically abusive episodes.

**Table 4:** Comparing proportions of abusive episodes committed by ICGs across ICG characteristics

Subtype of abuse	Psychological, n (%)			Physical, n (%)			Overall, n (%)		
	No abuse	Abuse	*P*^a^	No abuse	Abuse	*P*^a^	No abuse	Abuse	*P*^a^
**ICG characteristics**									
Gender									
Female	124 (35.0)	230 (65.0)	0.320	323 (91.2)	31 (8.8)	0.422	109 (32.2)	229 (67.8)	0.355
Male	66 (39.5)	101 (60.5)		146 (89.0)	18 (11.0)		59 (36.4)	103 (63.6)	
Education									
Primary school	23 (44.2)	29 (55.8)	0.233	51 (100.0)	0 (0.0)	**0.044**^**b**^	22 (44.0)	28 (56.0)	0.164
High school	52 (42.3)	71 (57.7)		113 (90.4)	12 (9.6)		46 (39.0)	72 (61.0)	
Vocational school	45 (36.0)	80 (64.0)		105 (88.2)	14 (11.8)		38 (32.8)	78 (67.2)	
University/college ≤4y	39 (34.2)	75 (65.8)		106 (92.2)	9 (7.8)		35 (31.3)	77 (68.8)	
University/college >4y	32 (29.6)	76 (70.4)		95 (87.2)	14 (12.8)		28 (26.7)	77 (73.3)	
Relationship with PWD									
Spouse/cohabitant/partner	83 (25.2)	246 (74.8)	**0.000**	274 (85.1)	48 (14.9)	**0.000**^b^	71 (22.8)	240 (77.2)	**0.000**
Child	93 (54.4)	78 (45.6)		170 (99.4)	1 (0.6)		82 (50.0)	82 (50.0)	
Other	16 (64.0)	9 (36.0)		24 (100.0)	0 (0.0)		15 (62.5)	9 (37.5)	

^a^Pearson’s chi-squared test, exception: ^b^Fisher’s exact test

**Table 5:** Comparing proportions of abusive episodes committed by ICGs with subtypes of PWD aggression

Subtype of abuse	Psychological, n (%)			Physical, n (%)			Overall, n (%)		
	No abuse	Abuse	*p*^a^	No abuse	Abuse	*p*^a^	No abuse	Abuse	*p*^a^
**Subtype of PWD aggression**									
Psychological									
No aggression	169 (49.6)	172 (50.4)	**0.000**	306 (91.1)	30 (8.9)	0.618	150 (46.6)	172 (53.4)	**0.000**
Aggression	21 (11.7)	158 (88.3)		157 (89.7)	18 (10.3)		16 (9.4)	155 (90.6)	
Physical									
No aggression	186 (39.2)	289 (60.8)	**0.002**	429 (91.9)	38 (8.1)	**0.001**^b^	164 (36.4)	286 (63.6)	**0.001**
Aggression	6 (14.6)	35 (85.4)		29 (72.5)	11 (27.5)		4 (10.5)	34 (89.5)	
Overall									
No aggression	152 (49.8)	153 (50.2)	**0.000**	280 (91.5)	26 (8.5)	0.170	141 (47.6)	155 (52.4)	**0.000**
Aggression	23 (13.5)	147 (86.5)		148 (87.6)	21 (12.4)		18 (10.8)	149 (89.2)	

*PWD* Person with dementia

^a^Pearson’s chi-squared test, exception: ^b^Fisher’s exact test

## Discussion

This study aimed to describe the proportion of abusive episodes among home-dwelling persons with dementia and their ICGs and to explore differences between ICGs who have committed abusive acts toward the person with dementia and those who have not. The results show that two-thirds of ICGs reported at least one abusive episode in the past 12 months, and approximately one-third had experienced aggression from the person with dementia. We also found that the proportion of abusive episodes from ICGs to the person with dementia is higher if the ICG is a spouse/partner of the person with dementia and if they experience aggression from the person with dementia.

### Occurrence of abusive episodes

In our study, we found that 66.3% of ICGs reported that they had committed at least one abusive act toward the persons with dementia in the past year. This is more than four times higher than the estimate of 15.7% among community-dwelling older persons [2], and slightly higher than the highest prevalence found in previous studies of older persons with dementia (62.3%) [6]. When considering the increased risk of abuse for persons with dementia [6], it is reasonable that the occurrence in our study is higher than the prevalence among cognitively healthy older persons found by Yon et al. [2]. The COVID-19 pandemic may also have contributed to an increase in the occurrence of abusive episodes in the present study, partly due to increased strain on ICGs. Among 35,143 Norwegian registered nurses, 36% reported an increased burden for ICGs during the pandemic, and it was mostly patients receiving homecare services who lost important respite care and support services [33].

Psychological abuse was the most common subtype of abusive episodes in our study, with 63.5% reporting at least one episode in the past year. Several previous studies investigating two or more subtypes of elder abuse in the same setting also found psychological abuse to be most common [8, 11, 12, 34–36], ranging from 33 to 62.3%. In a study from Florida, US, VandeWeerd et al. [13] found that 60.1% of ICGs used verbal aggression against the person with dementia. The occurrence in our study is higher than in these studies. The studies vary in measurements used and timeframe: the study with the lowest prevalence [34] used a score of at least 2 as a threshold for abuse, and the study with the highest prevalence [35] used any abusive behavior in the past month as the threshold for abuse. In the present study, we used at least once in the past year as the threshold, but the proportion of psychological abuse would still be quite high if a criterion of two or more episodes was applied. Looking only at “shouted or yelling at the person with dementia” in Table 2 and 47% reported doing this at least two times in the past year.

The same studies that found psychological abuse to be most common, reported a prevalence of physical abuse ranging from 4 to 20% [8, 11, 12, 34–36]. In the present study, 9.4% of the ICGs reported some form of physical abuse in the past year. Globally the prevalence is estimated to be 2.6% among the general older population [2]. The occurrence in the present study is almost four times higher than the global estimate, which was expected since persons with dementia are at a higher risk of abuse than older persons in general. The present study has less than half the occurrence of physical abuse as the study with the highest prevalence (20%) [11]. One reason could be that Cooney et al. only included ICGs who lived with the persons with dementia, and in the present study, 35% of the participants did not co-reside with the person with dementia. Co-residency between the ICG and the person with dementia increases the risk and occurrence of abusive episodes [6].

In the present study, 3.9% reported some form of episodes related to financial abuse. To our knowledge, this is the first survey study among ICGs of home-dwelling person with dementia to explore financial abuse. Globally, a prevalence of 6.8% is estimated [2]. The occurrence in the present study is almost half of the global estimate. We speculate whether the low incidence in our study may be related to the fact that Norway is a rich welfare state with good access to help from the public sector, which makes relatives less financially dependent on others. Sandmoe et al. [3] found a prevalence of 0.6% in the past year reported by home-dwelling older persons without cognitive impairment in Norway, which is even lower.

To our knowledge, this is the first survey study exploring the occurrence of ICG-reported sexual abuse among home-dwelling persons with dementia. This might be because of the sensitive and private nature of sexuality [6]. We also found it difficult to ask ICGs with various relationships to the person with dementia about such a sensitive matter without risking withdrawal of participation. After consulting with health care personnel with extensive experience in guiding ICGs, we chose questions about sexual abuse that did not ask directly about violent sexual assaults, but instead focused on ICGs doubts about consent and possible inappropriate actions (see Table 2 for items). Our study demonstrated that 2.4% of ICGs reported sexual abuse. The majority was related to doubts about willingness or consent from the person with dementia. Sandmoe et al. [3] found that 0.5% of older Norwegian home-dwelling persons reported sexual abuse in the past year. The global prevalence is estimated to be 0.9% [2]. It is hard to draw parallels between the occurrence in the different studies, especially because the questions in the present study were study-specific. However, our results show that some ICGs experience doubts concerning their sexual relations with the person with dementia, which health care personnel should be aware of and be open to discuss with ICGs and persons with dementia.

The global estimate for neglect of older persons is 4.2% [2]. In the present study, 6.5% of ICGs reported neglect of the person with dementia, which is surprisingly close to the global estimate. Because of the increased risk of abuse among persons with dementia, we would expect the occurrence in the present study to be higher. Pickering et al. [37] found that 50% of ICGs who co-resided with the person with dementia reported at least one neglectful behavior within 21 observation days. In our study, between 34 and 74.6% of the ICGs answered “not applicable” on the different neglect items, indicating either that the person with dementia did not need help with those tasks, or that someone else (formal carer or others) helped the person with dementia. This might be a contributing factor to the occurrence being lower than expected. One cannot neglect a task that one does not perceive a responsibility for.

### Characteristics of ICG reporting and not reporting abusive acts

The present study shows a significantly higher proportion of abusive episodes when the ICG is a spouse or partner compared to other relationships with the person with dementia for all subtypes of abuse. These results are supported by a review of elder abuse in the general older population where Pillemer et al. [4] found that spouses or partners were the most common perpetrator of abuse in Europe. These results were not conclusive because the authors also found a cultural difference, where similar results also were found in the US and Israel, whilst in Asia children and children-in-law were more likely to commit abuse. This may be a result of living arrangements and not their relationship per se. In the US and Europe, it is mostly spouses who co-reside with older persons, while in Asia it is more common that children or extended family share the same household as older persons [38]. Several studies have found that the number of co-residing days or co-residency is a risk factor for abuse among persons living with dementia [35, 39–41]. According to a national ICG survey in Norway, spouses/partners are among those who spend most hours on caregiving, and the perceived caregiver burden is associated with the extent of caregiving tasks [42]. During the COVID-19 pandemic, there was an increase in informal care, especially among ICGs who co-resided with the person with dementia [20]. There is also evidence that BPSD increased during the pandemic [21]. High levels of burden and high levels of BPSD are both known risk factors for abuse [12, 35, 43, 44]. Hence, the situation during the pandemic might have reinforced the association between abusive episodes and the ICG being a spouse/partner.

The test for the association between ICGs’ educational level and physical abusive episodes showed a significantly (*p* < 0.05) higher proportion of abuse among ICGs at all other educational levels compared to ICGs with only a primary school education. Although the results within psychological and overall abuse were not significant (*p* > 0.1), the occurrence of abusive episodes increased with a higher level of education. Botngård et al. [27] and Malmedal et al. [45] found similar results among health care personnel in Norwegian nursing homes. The authors speculated that higher education made health care personnel better at reflecting and more critical of their practices; they, therefore, identified more of their behavior as potentially abusive. It is difficult to draw parallels with ICGs directly from health care personnel. Different studies have shown diverging results regarding the sociodemographic characteristics of perpetrators, including an increased risk of neglect with lower education or no significant differences at all [46]. This may be due to unknown confounding factors not controlled for in our analysis.

In the present study, the proportion of ICGs who had committed abusive episodes was significantly higher among those who had experienced aggression from the person with dementia compared to those who had not. Several previous studies have identified aggression as a risk factor for ICG abuse [7, 8, 13, 14, 36, 47]. VandeWeerd et al. [13] found that ICGs who experienced verbal aggression from their care recipient had an eight times higher risk of being verbally aggressive themselves. There was a four times higher risk of an ICG being physically abusive if the person with dementia was acting physically abusively [14]. In the present study, the proportion of abusive episodes is higher in all subtypes when the person with dementia is aggressive toward the ICG. When it comes to physical abuse, this association is only statistically significant if the aggression is physical. These results imply that the risk of the ICG being abusive is higher when they experience aggression. However, within the physical subtype, the increase in risk only applies if the aggression from the person with dementia also is physical. Further study may illuminate the reasoning why this risk only applies to physical aggression, for instance, that it was an act of self-defense. In the present study, only 7.6% of the ICGs reported physical aggression, but it is estimated that more than 20% of ICGs at some point experience severe aggression from the person with dementia [48]. The reason why the proportion is lower in our study is probably because several of the persons with dementia are in the early stages of the disease. In any case, aggression from the person with dementia makes the distinction between perpetrator and victim unclear, alternating, and bi-directional in some circumstances. Although the ICG seemingly has the upper hand in the relationship due to them being cognitively healthier, they can be exposed to unpleasant, threatening, and violent episodes. Therefore, for the safety and wellbeing of both the person with dementia and the ICG, it is important that formal caregivers such as health care personnel help the ICG to develop coping skills and provide preventive interventions to avoid physically aggressive behavior from the person with dementia.

### Strengths and limitations

The present study has several strengths. Firstly, the sample size is large compared to many similar studies [11, 34–36, 43, 49], particularly in relation to the population of Norway. Only a few studies in the same type of population/setting have had similar sample sizes [12, 50]. Secondly, the sample includes ICGs from all Norwegian regions and persons with dementia living in both urban and rural areas. Thirdly, to our knowledge, this is the first survey among ICGs of persons with dementia that measures all subtypes of elder abuse.

The present study has some limitations that must be considered when interpreting the results. Firstly, we only used ICGs as informants. In addition to possible recall bias, they may be reluctant to report abusive episodes because of stigma, shame, or fear of consequences if cases of abuse are revealed. Secondly, the data collection was carried out during the COVID-19 pandemic. Restrictions and changes in services during the pandemic may have influenced the occurrence of abusive episodes.

Thirdly, it is difficult to compare the prevalence or occurrence of abuse across different studies because there is no consensus or gold standard for measuring incidences of abuse or abusive episodes [6]. Many factors contribute to variation in prevalence between studies and regions, such as sample size, sampling methods and measures, culture-specific norms and expectations, and definitions of elder abuse [2]. In the present study, no threshold or criterion of abuse has been applied and all abusive episodes are treated equally in the analysis. Therefore, caution must be taken when comparing occurrences with other studies regarding elder abuse.

Fourthly, we could not randomly select ICGs to participate because there is no overview of the total population of ICGs of persons with dementia in Norway. This, and the relatively low response rate (36.7%), increase the risk of bias in the study results. We have tried to mitigate this by applying several recruitment strategies including volunteer organizations, municipal health services, and specialist health services. The present study has a higher proportion of spouses/partners compared to the ICG population in general [42]. This might be because spouses/partners in general take on more care tasks than other ICGs and therefore are more likely to participate because they feel more strongly about the study theme. A higher proportion of spouses/partners might give a higher occurrence of abuse in the sample compared to the population because of the association between spouses and abusive episodes. On the other hand, ICGs with the heaviest burden are probably underrepresented in the sample. There are two main reasons for this: (1) heavily burdened ICGs might not find the time or energy to fill out the comprehensive questionnaire; and (2) health care personnel and volunteers with knowledge of the ICGs’ situation may have been reluctant to ask heavily burdened ICGs if they wanted to participate. We expect this to lower the occurrence in the sample, assuming there is an association between caregiver burden and abusive episodes.

## Conclusion

This study explores abusive episodes among home-dwelling persons with dementia and their ICGs in Norway. The results demonstrate that a majority of ICGs commit some form of abusive episodes toward the person with dementia, and acts within the scope of psychological abuse are most frequent. Although a minority, many ICGs also experience aggression from the person with dementia. ICGs contribute enormously to dementia care, but they also bear a heavy burden. This burden could contribute to abusive episodes, which may increase the persons with dementia’s behavioral symptoms and aggression towards the ICG, thus creating a negative spiral with increased abuse and increased burden where both the ICG and the person with dementia are suffering.

The results of this study have clinical implications and expands the knowledge about elder abuse and abusive episodes among home-dwelling persons with dementia. Health care personnel need to know the risks of abuse to take preventive measures in collaboration with the person with dementia and their ICG. Our findings are also important for policymakers when they plan and prioritize in relation to health and care services. Persons with dementia are a large group that require a great deal of both informal and formal care, and increasing prevalence is expected in the years to come. Further research is needed to better understand the complexities of elder abuse. In particular, there is a need for larger transnational studies focusing on risk and protective factors. It is critical to increase understanding of the dynamics of abuse occurring in these complex relationships to develop interventions that reduce risk and prevent abuse.

## Data Availability

The datasets generated and analyzed during the current study are not publicly available due to ethical restrictions. Due to the nature of this research, participants of this study were not asked to agree for their data to be shared publicly. Data might be provided from the corresponding author if granted approval from the Regional Ethics Committee for Medical Research (REC) in Mid-Norway.

## Abbreviations

ICG: informal caregiver
BPSD: behavioral and psychological symptoms of dementia
